# A deep learning model for CT-based kidney volume determination in dogs and normal reference definition

**DOI:** 10.3389/fvets.2022.1011804

**Published:** 2022-10-28

**Authors:** Yewon Ji, Hyunwoo Cho, Seungyeob Seon, Kichang Lee, Hakyoung Yoon

**Affiliations:** ^1^Department of Veterinary Medical Imaging, College of Veterinary Medicine, Jeonbuk National University, Iksan, South Korea; ^2^INGRADIENT Inc., Seoul, South Korea

**Keywords:** artificial intelligence model, renal volume, renal segmentation, computed tomography, canine

## Abstract

Kidney volume is associated with renal function and the severity of renal diseases, thus accurate assessment of the kidney is important. Although the voxel count method is reported to be more accurate than several methods, its laborious and time-consuming process is considered as a main limitation. In need of a new technology that is fast and as accurate as the manual voxel count method, the aim of this study was to develop the first deep learning model for automatic kidney detection and volume estimation from computed tomography (CT) images of dogs. A total of 182,974 image slices from 386 CT scans of 211 dogs were used to develop this deep learning model. Owing to the variance of kidney size and location in dogs compared to humans, several processing methods and an architecture based on UNEt Transformers which is known to show promising results for various medical image segmentation tasks including this study. Combined loss function and data augmentation were applied to elevate the performance of the model. The Dice similarity coefficient (DSC) which shows the similarity between manual segmentation and automated segmentation by deep-learning model was 0.915 ± 0.054 (mean ± SD) with post-processing. Kidney volume agreement analysis assessing the similarity between the kidney volume estimated by manual voxel count method and the deep-learning model was r = 0.960 (*p* < 0.001), 0.95 from Lin's concordance correlation coefficient (CCC), and 0.975 from the intraclass correlation coefficient (ICC). Kidney volume was positively correlated with body weight (BW), and insignificantly correlated with body conditions score (BCS), age, and sex. The correlations between BW, BCS, and kidney volume were as follows: kidney volume = 3.701 × BW + 11.962 (*R*^2^ = 0.74, *p* < 0.001) and kidney volume = 19.823 × BW/BCS index + 10.705 (*R*^2^ = 0.72, *p* < 0.001). The deep learning model developed in this study is useful for the automatic estimation of kidney volume. Furthermore, a reference range established in this study for CT-based normal kidney volume considering BW and BCS can be helpful in assessment of kidney in dogs.

## Introduction

Many studies have shown that kidney volume is an important parameter in the evaluation of renal diseases, as it is associated with renal function and disease severity (1, 2). Kidney volume is reported to be helpful as a biomarker of severity and progression in diseases, for example, decreasing volume in chronic kidney disease (3) and the increasing volume in polycystic kidney disease (4, 5) and acute kidney diseases such as acute proliferative glomerulonephritis, acute tubular necrosis and acute interstitial nephritis (6). Glomerular filtration rate (GFR) is also reported to correlate well with kidney volume (1).

Owing to its importance in the assessment of renal function and diseases, several methods have been proposed to predict kidney volume based on diagnostic imaging. One study proposed ultrasonographic determination of kidney volume in dogs using prolate ellipsoid geometric formulae (7), but several studies have reported that measuring kidney size using ultrasound causes errors and can be inaccurate or have poor reproducibility (8–11). The voxel count method based on computed tomography (CT) has been reported to be an accurate method for evaluating kidney volume (12–14). However, this method has limitations in the clinical field because it is time consuming, as it is performed by drawing a region of interest (ROI) on every cross-sectional image of kidney in the CT images. The number of images needed to be hand-drawn was ~40–70 per CT scan, though it varies with the size of the dog and the slice thickness.

Many studies in human medicine have proposed deep learning methods, such as convolutional neural networks, for renal segmentation and automatic volume estimation (15–20). As a recent advance of deep learning based analysis methods, convolutional neural networks (CNNs) have shown promising performance in medical imaging tasks such as image classification, detection, and segmentation (21). U-Net is a modified architecture “fully convolutional network (22)”, which works with high resolution images and yields precise segmentations compared to previous architectures showing good results on various biomedical segmentation applications (23–25). Recently, a novel architecture called UNEt Transformers (UNETR) was introduced which demonstrated superior performance over both CNNs and other transformer-based models (26).

There have been several studies on texture analysis and the development of machine learning algorithms for canine radiographs (27–29) and CT images (30) in veterinary medicine, but there is no published method to detect the kidney and determine its volume in dogs from CT images using deep learning models.

In human medicine, several studies have used CT images to demonstrate correlations between kidney volume, body weight (BW), and age (2, 12). In veterinary medicine, previous studies have examined the relationship between kidney length and BW using CT images (31) and the relationship between renal cortical thickness and BW using ultrasound (32), but none have evaluated the relationships between kidney volume, BW, and age using CT images. In addition, there is no established reference range for kidney volume measured from CT images in dogs. In spite of the importance of kidney volume assessment in clinical perspective, the lack of reference range for kidney volume estimated in CT images in normal dogs and the time-consuming nature of conventional voxel count method to evaluate kidney volume in CT images have been the impediments for clinicians to apply it.

In this study, we developed an automated volume estimation method for the veterinary field. The primary objective was to develop a deep learning model to detect the kidney and quantify its volume from CT scans of dogs. The second objective was to establish reference range values for kidney volume in normal dogs considering BW, body condition score (BCS), and age.

## Materials and methods

### Pilot cadaver experiment

To ensure the accuracy of the manual voxel count method, CT scans were performed on six *ex vivo* formalin-fixed cadaveric kidneys (Alexion, TSX-034A, Canon medical system Europe B.V., Zoetermeer, Netherlands). Imaging protocols were 120 kVp, 150 mAs, 512 × 512 matrix, and 1 rotation time with a 1 mm slice thickness. In total, 367 axial slices from 6 *ex vivo* kidneys were collected. The numbers of image slices segmented in each kidney are 66, 63, 46, 47, 85, and 60. The kidney volumes were calculated using the manual voxel count method from the CT images and compared with the actual volume which was measured using the water displacement method. The values obtained using the two methods were compared.

### Dataset

#### CT image acquisition and dataset for model development

A total of 386 CT scans (Alexion, TSX-034A, Canon medical system Europe B.V. and Zoetermeer, Netherlands, Revolution ACT, GE Healthcare, Milwaukee, WI, USA and Brivo CT385, GE Healthcare, Milwaukee, WI, USA) from 211 dogs randomly collected from multiple centers, for model development and performance testing. Imaging protocols were as follows; 120 kVp, 150 mAs, 512 × 512 matrix and 0.75 rotation time (Alexion),120 kVp, 84 mAs, 512 × 512 matrix and 1 rotation time (Revolution ACT) and 120 kVp, 69 mAs, 512 × 512 matrix and 1 rotation time (Brivo CT385). CT images with slice thickness of 1–2.5 mm were used. Iohexol (Omnipaque 300, GE Healthcare, Shanghai, China) at 750 mg iodine/kg, at a flow rate of 2.5–3.5 ml/s was injected.

Pre- and post-contrast CT images were included in the training and validation data for the performance tests. Dataset for training data and validation data was split as ratio of 80 to 20 considering the distribution of original dataset. Therefore, a total of 309 CT scans (179 pre-contrast and 130 post-contrast) were randomly chosen for the training data, and the remaining 77 CT scans (44 pre-contrast and 33 post-contrast) were used for validation. Thus, 182,974 image slices (107,340 axial slices, 36,818 coronal slices, and 38,816 sagittal slices) were used to develop the model. Dogs with both clinically normal and abnormal kidneys were included since the purpose of this model is focused on kidney detection and volume estimation. CT scans with motion artifacts, those without volume information, or that had any axis less than a certain size were excluded from this study because the model's input and output required a certain size and images less than this patch size could not be applied to training. Post-contrast CT scans in which the kidneys were not properly enhanced were also excluded. This study was approved by the Institutional Animal Care and Use Committee of Jeonbuk National University (approval no. JBNU 2021-0156).

### CT image acquisition and dataset to establish a normal reference range for kidney volume

CT scans of 159 normal dogs were used to establish a normal reference range for the kidney volume of dogs. The medical records of the dogs, including clinical data of sex, age, BW, and laboratory examination results, were also collected. The inclusion criteria for the normal group were normal blood analysis results, no abnormal findings on diagnostic imaging, or clinical signs associated with renal function.

### Manual segmentation and pre-processing

#### Manual segmentation

**Figure 1:**
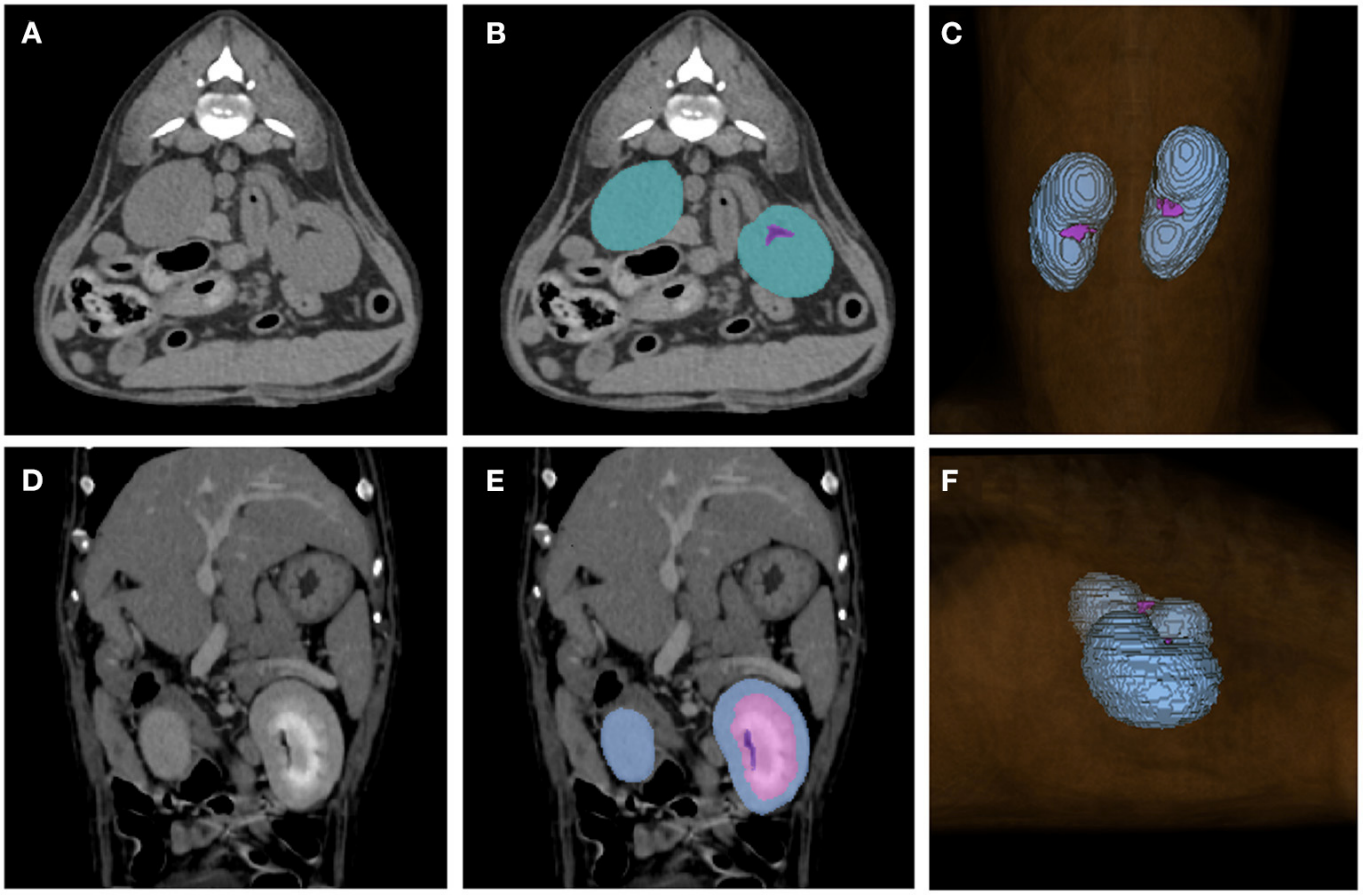
Examples of manual segmentations. Kidneys of pre-contrast **(A,B)**, post-contrast **(D,E)** images and the image showing final result of 3D segmentation **(C,F)** using the 3D-segmentation tool (Medilabel software) were manually segmented. In the pre-contrast images, kidneys were segmented into two classes: parenchyma (blue-green color in the labeled image) and fat around the renal pelvis (purple color in the labeled image). The post-contrast images were classified into three classes: cortex (Class 1, light blue color in labeled image), medulla (Class 2, pink color in labeled image), renal pelvis, and fat around the renal pelvis (Class 3, purple color in labeled image). When the segmentation for each single image is done, the 3D-segmentation is shown as **(C,F)**. The sum of the cortex and medulla was considered the parenchyma when the volume was calculated.

The CT scans included in the study were manually labeled by 10 clinicians (Residents in the Veterinary Medical Imaging Department of the Teaching Hospital of Jeonbuk National University) using Medilabel software (Ingradient,Inc., Seoul, South Korea), a 3D-segmentation tool. Both pre- and post-contrast scans were used for segmentation. In pre-contrast images, the renal parenchyma, renal pelvis, and the fat around it were segmented separately to avoid false training results where the model might recognize the fat around the renal pelvis as a part of the kidney (Figures 1A,B). Since cortex and medulla can be distinguished in post-contrast images, kidney was segmented into following three classes: 1. cortex, 2. medulla, 3. renal pelvis and the fat around it (Figures 1D,E). Then the sum of cortex and medulla was considered as the renal parenchyma, as it was in pre-contrast images. When the segmentation of each image is done, 3D segmentation of the kidneys is shown (Figures 1C,F).

#### Pre-processing

The training data was pre-processed in the following four steps: non-zero region crop, kidney localization, data resampling, and pixel normalization in PyTorch using TorchIO (33).

A non-zero region crop is the process of cropping out the background to exclusively obtain the actual region of interest. Voxels with certain values were considered as the background. The images were then divided into background and foreground images, and the image crop was performed so that the final image included a non-zero region without loss. The non-zero cropping can be performed by finding and cropping the largest contour on the sagittal and dorsal plane of the CT images.

Kidney localization was conducted to allow the deep learning model to train only the target region (kidney) without interfering structures in the images. To perform this processing automatically, we assumed that the kidney is located close to the caudal margin of the lung. Then, the lung can be roughly found by the average pixel intensity and histogram which is calculated from axial slices tracked from the head-sided of each dog. After detecting the caudal margin of the lung, the CT image could be cropped with a sufficiently large window to include the kidney in the cropped region. To avoid possible errors during these sequences, enough margin is added to the calculated values.

One of the impediments to model training is that the pixel spacing is different for each CT scan. Data resampling was performed to fix all the different pixel spacings to the same size (*x* = 1.5, *y* = 1.5, and *z* = 2.0).

Pixel normalization was performed to clip the minimum (−1,024) and maximum (3,025) Hounsfield unit (HU) values to minimum (−175), maximum (250).

### Deep learning model architecture and implementation details

#### Model architecture

**Figure 2:**
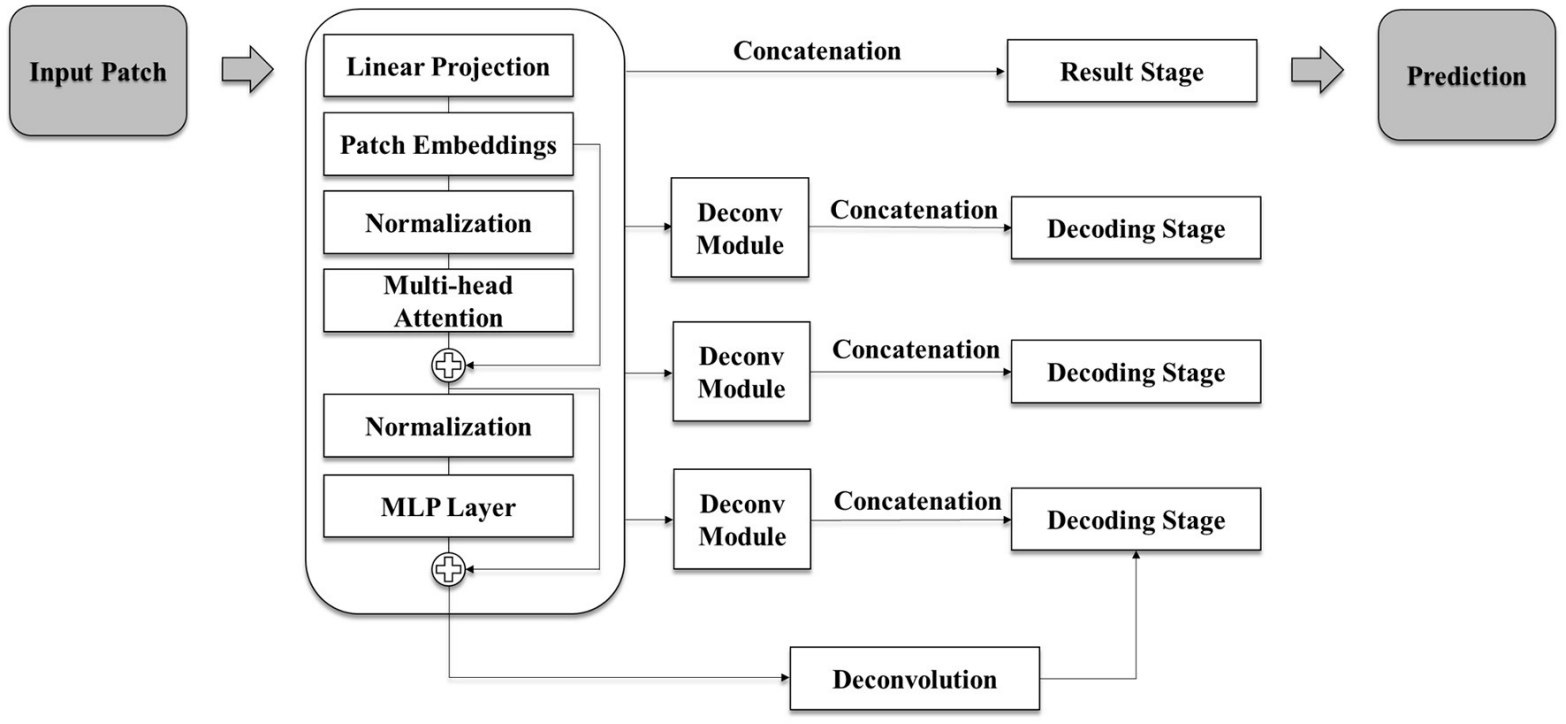
Schematic illustration of the model architecture used in this study. The model is based on UNETR architecture and has a segmentation network of encoder-decoder structure with 12 transformer blocks and three decoding stages. Each deconvolution module consists of repeating deconvolution and convolution, batch normalization, and activation functions. Each decoding stage is an up-sampling stage which conducts up-scaling after two rounds of 3D convolution, batch normalization, and activation function tasks.

Two recently proposed model architectures, i.e., nnUNet (34) and UNETR are compared for our experiment. In this study, an architecture based on UNETR (Figure 2) was used to auto-segment the kidney since UNETR showed better performance in the preliminary experiment. The model is a segmentation network that has an encoder-decoder structure with 12 transformer blocks and three decoding stages.

#### Implementation details

As the model predicts volume into 3 classes, the input channel of 1 and the output channel of 3 utilizes. To keep the features in the memory space of GPU, input volume size of (x = 96, y = 96, z = 96), hidden layer dimension of 768, feed-forward layer dimension of 3,072 and attention head number of 12 are used. For training, each random data augmentation is performed in probability of 0.1 and AdamW optimizer with initial learning rate of 0.00001 is used to minimize the loss function. For further details, the decoding stage of the model is also illustrated in Figure 2.

The PyTorch framework and Medical Open Network for AI (MONAI) were used to construct our model. Previous studies have shown that the combined loss function, i.e., weighted addition of cross-entropy and dice loss function, can improve the performance of the segmentation network (35–38). Due to our network performing segmentation on binary class and predicting various sizes of regions of interest, the combined loss function is utilized as a combination of the disc loss function and categorical cross-entropy (CE) loss function into the same scale.

Loss function is used to improve the performance of deep-learning models in spite of the class imbalance owing to the varying size of kidneys in the dataset. The Loss function used in this study is as follows: Loss function F = *Loss*_*dice*_ + *Loss*_*CE*_. *Loss*_*dice*_= 1 - $\frac{2\;*{\;}_{true}\;*\;Pixel_{pred}}{\sum {Pixel_{true}}^{2}+\sum {Pixel_{pred}}^{2}+\;\epsilon }$, *Loss*_*CE*_ = −∑*Pixel*_*true*_·log(*S*_*pred*_), respectively (*Pixel*_*true*_, 0 for the background, 1 for the kidney; *S*_*pred*_, sigmoid output (0~1); *Pixel*_*pred*_, 0 for the background, 1 for the kidney; pred, prediction).

Data augmentation was performed to elevate the performance using random crop, random flip, random rotation, and random intensity shift. Parameters for each transformations are selected to be realistic on the given images. The augmented images were also in distribution of original images which means the mean DSC of the model increases with the augmentation. Deep-learning model training was conducted for 400 epochs using an RTX A5000 GPU. The weight of the model showed the best validation DSC score during the training is selected as the final result of the model. The loss curve is monitored not to diverge during the training.

### Post-processing and volume measuring

#### Post-processing

To exclude unnecessary parts in our results, each prediction was divided into connected components, leaving four major components. Then, re-ordering was conducted using the voxel location data of each component and the Euclidean distances among the components. The component with the most information after the reordering process was selected for the final prediction.

#### Volume measuring from CT images using the voxel count method

The volume of the unit voxel in the CT images can be calculated by x-spacing ^*^ y-spacing ^*^ z-spacing, and the final volume prediction was performed using the counted number of voxels in the ROI after post-processing (39), i.e., x-spacing ^*^ y-spacing ^*^ z-spacing ^*^ number of voxels.

### Model accuracy and statistical analysis

Statistical analysis using the *t*-test and absolute agreement on intraclass correlation coefficient (ICC) were performed to evaluate the accuracy of the manual voxel count method when comparing the results between the water displacement method and manual voxel count method. For the normal distribution test, Kolmogorov-Smirnov test and Shapiro-Wilk test were performed. Levene's test for equality of variances was performed.

The Dice similarity coefficient (DSC) was used to evaluate the performance of the auto-segmentation of kidneys by deep-learning model (40, 41). DSC measures the relative voxel overlap between segmentations performed by the automated model and the manual segmentations so that it shows the similarity between segmentations performed by automated model and the manual segmentations. DSC close to 1 implies higher similarity between two segmentations. The DSCs showing the similarity between the automated model and the manual segmentations of whole kidney, cortex and medulla are measured. The DSC was measured using the following formula:

DSC = $\frac{2\;TP\;}{2\;TP+FP+FN}$ (TP, true positive; FP, false positive; FN, false negative).

The total kidney volume measured by the deep learning model and the manual voxel count method were compared to evaluate the accuracy of the deep learning model. Seventy-seven test sets randomly chosen for the validation data that were not used as training data were used. Statistical analysis was performed using Lin's concordance correlation coefficient (Lin's CCC) and ICC.

One-way ANOVA was used to show the difference of kidney volume/BW indices among the following four groups; Intact males, neutered males, intact females, and neutered females. Kolmogorov-Smirnov test and Shapiro-Wilk test were performed for the normal distribution test. Mann-Whitney test was used to show the difference of volume/BW indices and kidney volume between intact vs. neutered dogs and male vs. female dogs. The Pearson correlation coefficients, multiple regression analyses are used to show the correlations between BW, BCS, age and kidney volume. The volume/BW index was used to normalize kidney volume by BW. More detailed information is given at the respective result sections.

Statistical significance was set at *P* < 0.05. Statistical analyses were performed using SPSS version 27.7 (SPSS Inc., Chicago, IL, USA).

### Time measurement

The approximate time consumed for segmentation per dog was counted from the start and to the end of labeling using the Medilabel software (Ingradient Inc., Seoul, South Korea) and the time consumed for auto-segmentation by deep-learning model was recorded.

## Results

### Pilot cadaver experiment; good agreement between manual voxel count method and actual volume

Volumes measured by the manual voxel count method and water displacement test showed good agreement, with an ICC of 0.997 [95% CI:0.401–1.00, *p* < 0.001]. The Kolmogorov-Smirnov test showed satisfaction of normality assumption of two groups (*p* = 0.059, *p* = 0.128, manual voxel count method and water displacement method, respectively), so did the Shapiro-Wilk test of *p* = 0.104 and *p* = 0.210, respectively. Levene's test showed equality of variances (*p* = 0.990). The t-test showed no statistically significant difference between the two methods (*p* = 0.864).

### DSC and the time consumed for automatic estimation of kidney volume; deep learning model shows high similarity compared to manual segmentation in a short time

Ninety eight neutered males, 12 intact males, 79 neutered females, and 22 intact female dogs were included in the study. The mean BW of the dogs was 6.83 kg (range: 1.48–41.5 kg), mean age was 9.6 years (range: 0.3–20 years).

**Figure 3:**
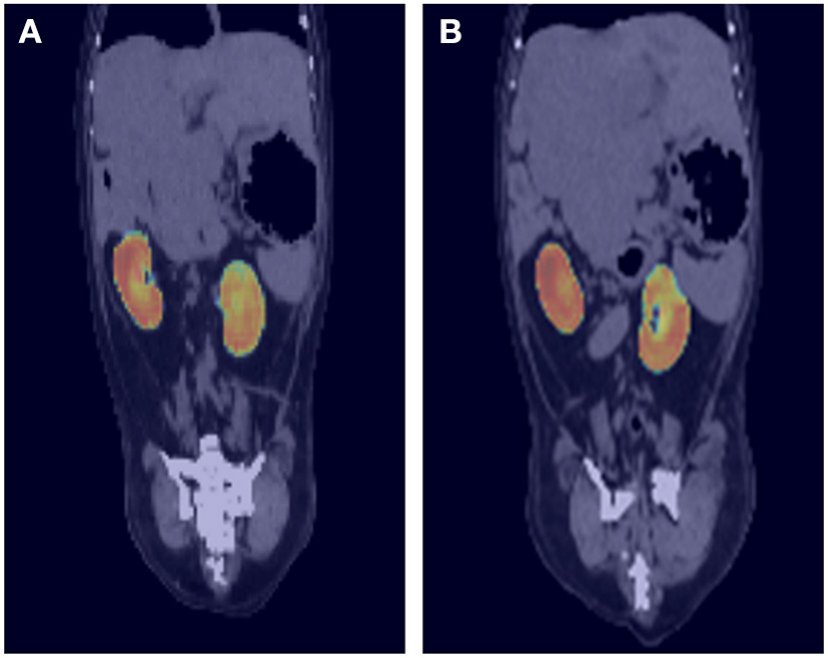
Probability maps overlaid on ground truth computed tomography (CT) images. The output of the deep-learning model at each location is generated as a probability map and expressed as a heat map. For example, image **(A)** shows light blue to green color at the cranial border of right kidney and image **(B)** also shows similar color at the cranial border of left kidney. As the borderline between right kidney and liver is not usually delineated well on pre-contrast CT images, the probability is low, and it is expressed as blue to green color rather than red.

To evaluate the performance of the model, the DSC comparing the result of manual segmentation and automated segmentation was calculated for the 77 test sets. The overall mean DSC values before post-processing and after post-processing were 0.912 ± 0.057 and 0.915 ± 0.054 (mean ± SD), respectively. Figure 3 shows the corresponding CNN-generated probability maps in color.

When the cortex and medulla were predicted separately, the overall mean DSC values were 0.772 ± 0.152 and 0.779 ± 0.143 for the cortex and medulla, respectively. Moreover, to evaluate the combined loss function, the network is trained with three different loss function while the training strategy is fixed equally. The network showed 0.886 of DSC with only cross-entropy loss function and 0.869 of DSC with only dice loss function. Therefore, the result from the combined loss function mentioned above is used for volume prediction.

The mean time consumed for the automatic estimation of kidney volume in the validation set was 17.69 s per dog, while the manual segmentation method took ~30–45 min per dog.

### Total kidney volume agreement analysis; high similarity between deep learning model and manual voxel count method

**Figure 4:**
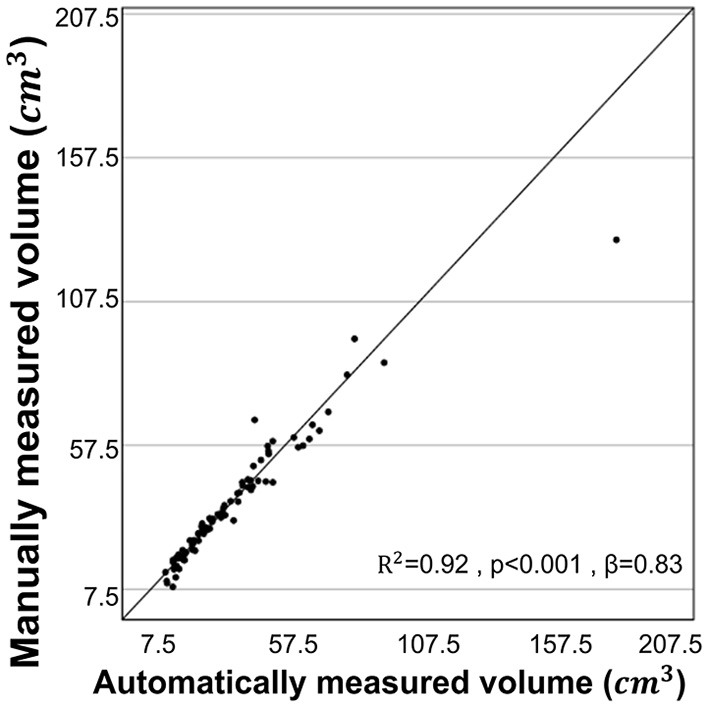
Correlation for volume measurements by manual voxel count method and deep-learning model. The volume estimated from the manual voxel count method and the automatically estimated volume by the deep learning model showed high correlation (r = 0.960, *R*^2^ = 0.92, *p* < 0.001).

The statistical results of the total kidney volume agreement between the deep learning model and manual voxel count method are summarized in Table 1, which shows that the results from both methods were similar. There is a substantial strength of correlation with Lin's CCC of 0.95 [95% CI:0.93–0.97]. The ICC of absolute agreement between the two methods was 0.975 (95% CI:0.961–0.984, *p* < 0.001). Figure 4 shows the high correlation between the two methods. The mean difference index (|*Ground truth volume*−*Automated volume*| / Ground truth volume) between the two methods was 0.081 and the estimated volumes measured from the pre- and post-contrast images were similar.

**Table 1:** Volume agreement analysis between the automatically estimated volume and the volume measured by the manual voxel count method.

**Volume agreement analysis**				
Auto vs. Manual	Lin's CCC	95% CI	ICC	95% CI
	0.95	[0.93, 0.97]	0.975	[0.96,0.98]

Auto, automatically estimated volume; Manual, volume measured by the manual voxel count method; CCC, concordance correlation coefficient; CI, confidence interval; ICC, intraclass correlation coefficient (absolute agreement).

### Kidney volume in normal dogs; formula established for the estimated kidney volume in normal dogs considering BW and BCS

The mean age of normal dogs included was 9.31 ± 4.07 years (mean ± SD) and mean BW was 6.78 ± 5.40 kg (mean ± SD). The sex distributions of the dogs included in this study were: 11 intact males, 69 neutered males, 15 intact females, and 64 neutered females. Mean age of male dogs and female dogs were 8.92 ± 3.99 years, 9.69 ± 4.05 years (mean ± SD), respectively. Mean BW of male dogs and female dogs were 7.39 ± 6.48 kg, 6.16 ± 3.98 kg (mean ± SD), respectively.

**Figure 5:**
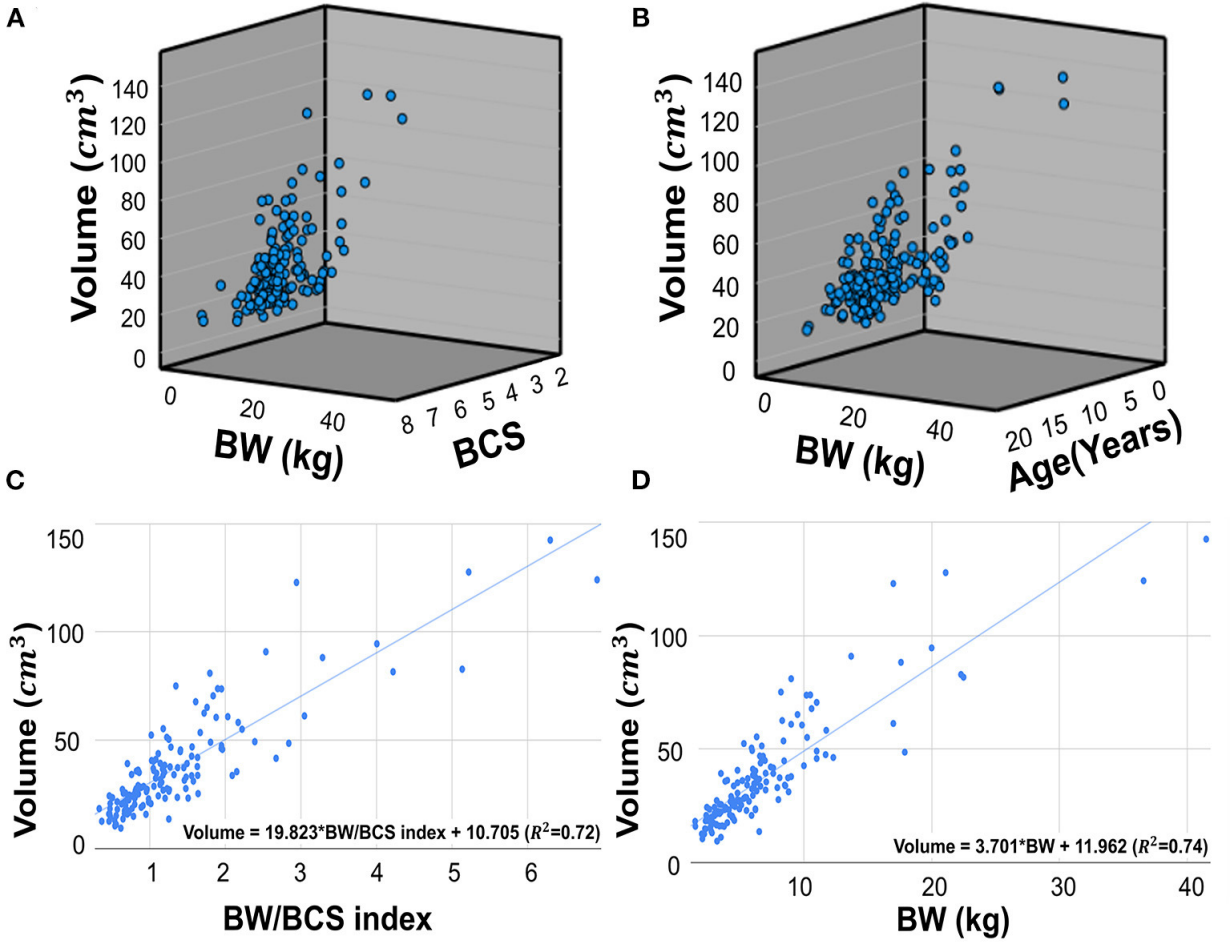
Scatter plots of multiple regression analysis and regression lines. Scatter plots of multiple regression analysis and regression lines. Multiple regression analysis of the relationship between BW, BCS, and kidney volume **(A)**. The regression equation is as follows: Kidney volume = 3.740*BW – 1.071*BCS + 17.181. (*R*^2^ = 0.74, *p* < 0.001 for BW and p=0.300 for BCS). Multiple regression analysis of the relationship between BW, age, and kidney volume **(B)**. The regression equation is as follows: Kidney volume = 3.697*BW – 0.054*Age + 12.497 (*R*^2^ = 0.74, *p* < 0.001 for BW and *p* = 0.818 for age). Scatter plots of regression analysis between kidney volume and BW/BCS index **(C)**. The regression equation is as follow: Kidney volume = 19.823 * BW/BCS index + 10.705 (*R*^2^ = 0.72, *p* < 0.001), The regression analysis between kidney volume and BW **(D)**. The equation is as follows: Kidney volume = 3.701*BW + 11.962 (*R*^2^ = 0.74, *p* < 0.001). BW, body weight; BCS, body condition score.

The mean kidney volume measured by manual voxel count method was 37.05 ± 23.16 *cm*^3^ (mean ± SD). The Pearson correlation coefficients (r) of the kidney volume between BW and the BW/BCS index (BW/BCS) were 0.860 (*p* < 0.001) and 0.849 (*p* < 0.001), respectively. Two multiple regression analyses were conducted with the first multiple regression analysis performed using BW and BCS as independent variables, and kidney volume as the dependent variable. This analysis found that kidney volume and BW were positively correlated, while BCS was negatively correlated. However, the negative correlation between kidney volume and BCS was not significant. This relationship is expressed in a regression equation as follows: kidney volume = 3.740 × BW – 1.071 × BCS + 17.181 (*R*^2^ = 0.74, *p* < 0.001 for BW and *p* = 0.300 for BCS) (Figure 5A). The relationship between kidney volume and BW/BCS was described as follows: kidney volume = 19.823 × BW/BCS index + 10.705 (*R*^2^ = 0.72, *p* < 0.001) (Figure 5C). The second multiple regression analysis was performed similarly to the first analysis, but with BW and age used as independent variables. The relationship is expressed by the following formula: kidney volume = 3.697 × BW – 0.054 × age + 12.497 (*R*^2^= 0.74, *p* < 0.001 for BW and *p* = 0.818 for age) (Figure 5B). The relationship between kidney volume and BW was expressed using the following formula: kidney volume = 3.701 × BW + 11.962 (*R*^2^ = 0.74) (Figure 5D).

**Figure 6:**
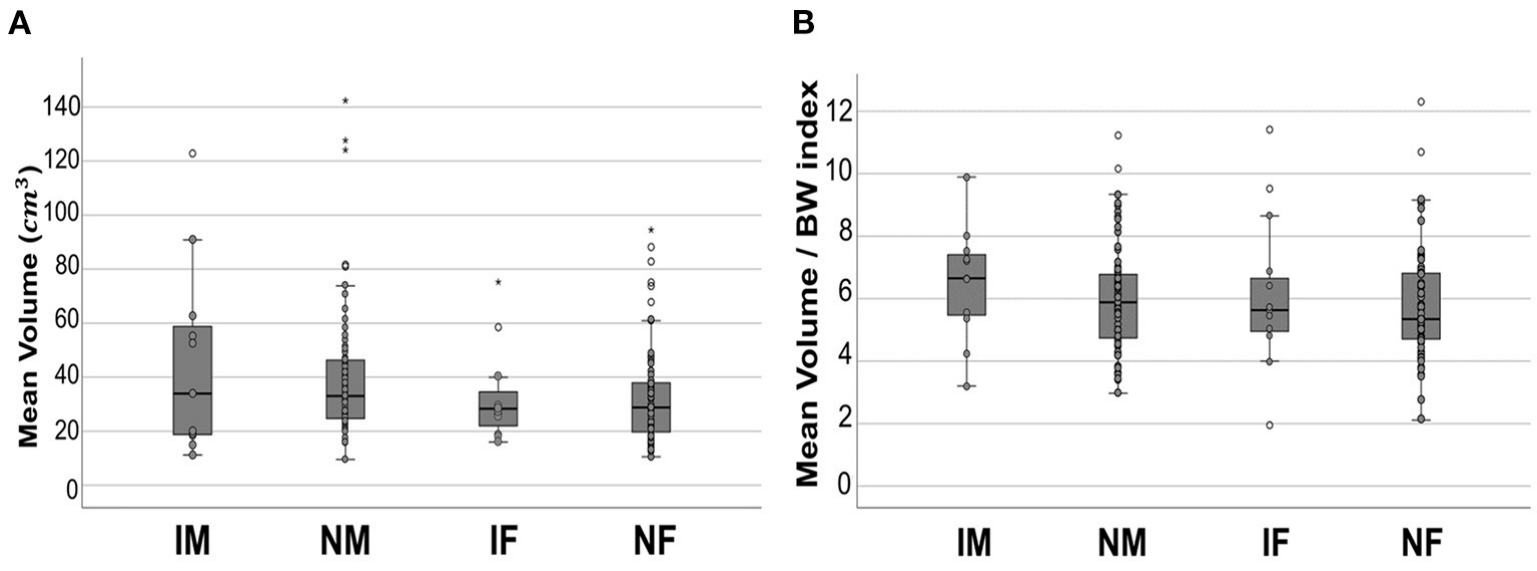
Boxplot of mean kidney volume and mean volume/BW indices. Mean kidney volume **(A)** and mean volume/BW indices **(B)** of each group. In both results, no statistically significant difference was found between the groups. All the data points are shown in the plot. The upper and lower edges of the box represent 25th (Lower quartile, Q1) and 75th (Upper quartile, Q3) percentiles. The vertical line (whiskers) between lower and upper extreme on each box represents the distribution range of data. The mild outliers (empty circles) are data points located outside of the whiskers, below Q1 – 1.5 × Interquartile range (IQR) or above Q3 + 1.5 × IQR. The extreme outliers (asterisks) are data points more extreme than Q1 – 3 × IQR or Q3 + 3 × IQR. IM, intact male; NM, neutered male; IF, intact female; NF, neutered female.

The mean age, BW, kidney volume, and volume/BW indices of the intact males, neutered males, intact females, and neutered females are summarized (Table 2). The mean kidney volume and volume/BW indices for each group are shown in a box plot (Figure 6). One-way ANOVA showed no statistically significant difference in volume/BW indices among the four groups (*p* = 0.658). Mann-Whitney test of volume/BW indices between intact dogs vs. neutered dogs (*p* = 0.379), and male dogs vs. female dogs (*p* = 0.256) were not significantly different. Meanwhile, the Mann-Whitney test showed significantly larger mean kidney volumes of male dogs than that of and female dogs (p = 0.042), 40.94 ± 26.43 and 33.10 ± 18.47 (mean ± SD), respectively.

**Table 2:** Data of the dogs in each group including number, mean age, mean BW, mean kidney volume, and mean volume/BW.

**(Mean ±SD)**	**Intact male**	**Neutered male**	**Intact female**	**Neutered female**
Number of dogs	*n* = 11	*n* = 69	*n* = 15	*n* = 64
Age (years)	7.04 ± 6.61	9.50 ± 3.66	9.49 ± 4.06	10.11 ± 4.10
BW (kg)	6.90 ± 4.92	7.58 ± 6.53	5.68 ± 2.85	6.1 ± 3.98
Volume (*cm*^3^)	45.61 ± 35.77	40.20 ± 25.10	31.69 ± 16.34	33.43 ± 19.18
Volume / BW	6.51 ± 1.85	6.01 ± 1.72	6.06 ± 2.35	5.80 ± 1.79

BW, body weight.

## Discussion

This is the first study in the veterinary medical field which presents an automatic method to segment kidneys using CT images in dogs and investigates the accuracy and precision of its segmentation and volume measurement using a deep-learning algorithm. Up until now, manual segmentation methods have been commonly used to measure kidney volume using computed tomography (CT) images. Although this method is accurate, it is time-consuming for clinicians and it is suggested that this automated method will help clinicians measure kidney volumes with significantly less work.

The *ex vivo* cadaver kidney experiment showed excellent agreement between the volume measured using the water displacement method and the manual voxel count method. Thus, the accuracy of the manual voxel count method used in this study was confirmed, as in previous studies. The volume measured by the manual voxel count method was not significantly different to the volume measured by the automated model, therefore it is assumed that the actual volume was similar to the volume measured by the automated model.

In this study, we developed a kidney detection model that showed 0.912 ± 0.057 and 0.915 ± 0.054 (mean ± SD) of DSC, pre-processing, and post-processing, respectively. Previous attempts have been made to develop machine learning methods to automatically segment kidneys in the clinical medical imaging field. da Cruz et al. (19) reported 0.96 of average DSC in final segmentation result, Daniel et al. (15) reported 0.93, and Thong et al. (20) reported 0.95 (Left kidney), 0.93 (Right kidney) by ConvNet-Coarse, 0.94 (Left kidney), and 0.93 (Right kidney) by ConvNet-Fine. Despite these promising results, this study has several limitations. Compared with the results achieved in the clinical medical imaging field, the model in our study showed slightly lower DSC results. Dogs have a wide range of body sizes, with the various breeds and this was an obstacle in the training process and a factor of the lower DSC compared to other models developed for humans because people have a relatively narrow body size range compared to dogs. In addition, the mean DSC for predicting the cortex and medulla was lower than that of the whole kidney. This could be attributed to the fact that the degree of contrast enhancement and its phase were not identical in each CT scan. In addition, segmentation was performed using the hand-drawn method, which could have subtle errors owing to the minimal pixel size of the segmentation tool. Further studies training the CT scans of the same enhancement phase can possibly increase the DSC of the cortex and medulla.

The time consumed estimating the volume of kidneys from CT images with the deep learning model in this study was significantly faster than the manual voxel count method. It is suggested that measuring kidney volume using this model is fast and just as accurate as the manual voxel count method, which can result in time saved for clinicians.

The model architecture in this study is a UNETR model based on a vision transformer which shows good performance in recognizing the anatomical structures of humans in a global context (26, 42). In the first attempt to build an architecture based on a 3D convolution-based model with the same data used in this study, the mean DSC obtained from the test sets was 0.84, which was unsuitable for the final result of this study. Thus, an architecture based on the UNETR was used to develop a better-performing model. Consequently, in this study the mean DSC (0.91) was significantly higher than the score obtained from the 3D convolution-based model. Considering the better result achieved by applying the UNETR model in veterinary medical imaging, further models developed for other organs in the veterinary field using this method are expected.

In this study, the BW and BW/BCS index were significantly positively correlated with kidney volume, which is consistent with the results of previous studies showing a significantly positive linear relationship between BW, BW/BCS index, and kidney size (32, 43, 44). In a previous study (32), the BW/BCS index showed a stronger correlation with renal cortical thickness than BW. However, our correlations between kidney volume and BW/BCS index and BW were not significantly different, even though BW had a positive correlation and BCS a negative correlation. The BCS of the dogs included in this study was mostly between four and six and dogs with extreme BCS were not included. It is assumed that this could be the reason that the BW/BCS index and BW showed similar correlations with kidney volume. It is more accurate to use the BW/BCS index in dogs with extreme BCS to calculate their normally expected volume; however, using BW alone can also offer similarly accurate results in dogs with normal BCS.

Our results showed that age was insignificantly correlated with kidney volume, regardless of sex. This might be associated with the inclusion criteria for dogs in this study. Dogs with clinically abnormal kidneys were excluded; therefore, only dogs with clinically normal kidneys were used to establish a normal volume range and investigate the relationships between kidney volume and several factors. Previous studies have reported on the relationship between age and kidney size in dogs, finding aging to have no significant effect on kidney size (44, 45). Several studies have shown that kidney parenchymal volume tends to decrease with age (46, 47). Conversely, some studies have found no significant evidence of a decline in kidney volume with age, except in very elderly patients (48, 49). Previous clinical studies have shown that imaging studies which exclude people with comorbidities tend to show less of a decline in kidney volume with age, whereas studies with less exclusion of comorbidity report a decline. In particular, a study of potential kidney donors did not show any evidence of decline (46). Considering these results, large sample of abnormal dogs with less exclusion and with an older age may show a greater decline in kidney volume with aging.

In this study, normal dogs were sorted into groups to determine whether sex and neutering were associated with a difference in normal kidney volume. We found no statistically significant influence of sex or neutering status on the volume/BW index among all groups. This was consistent with previous studies that found that sex had no significant effect on kidney size on radiographs in dogs (44) and cats (50). Meanwhile, when BW was not considered, the mean kidney volume in the male group was significantly larger than that in the female group, but there was no significant difference between male and female dogs when BW was considered and BW was strongly positively correlated with kidney volume. This is assumed to be associated with the larger mean BW in the male group compared with that of the female group.

Neutering status is known to be associated with the kidney size in animals. Neutered cats have smaller kidneys than intact cats (50). One study demonstrated hypertrophy of renal tissues as one of the effects of exogenous testosterone injection in mice, and hormonal influence is considered to be a reason for the smaller kidneys in neutered animals (51).

One of the limitations of this study was that the data used for the development of the deep learning model were not controlled, due to the retrospective nature of the study. A prospective study with controlled data could result in the development of a model with better performance. In addition, the model developed in this study was not capable of detecting lesions and had low accuracy in the detection of the cortex and medulla. Further studies are needed to develop a deep learning model that can detect lesions and the more detailed anatomical structures of the kidney. In addition, given that the wide range of body sizes of dogs was considered the main obstacle to model development in this study, adding more data for training could probably increase the model's accuracy and precision to overcome this factor.

In conclusion, the deep learning model developed in this study can potentially help clinicians easily estimate kidney volume from CT images in dogs. Furthermore, this study provides a reference range for kidney volume in normal dogs measured from CT images considering BW and BCS which can be applied to the clinical evaluation of kidneys.

## Data availability statement

The original contributions presented in the study are included in the article/supplementary material, further inquiries can be directed to the corresponding author.

## Ethics statement

The animal study was reviewed and approved by Institutional Animal Care and Use Committee of Jeonbuk National University (Approval No. JBNU 2021-0156). Written informed consent was obtained from the owners for the participation of their animals in this study.

## Author contributions

YJ and HY: conception, design, and drafting. YJ, HC, and HY: acquisition of data. YJ, HC, SS, KL, and HY: analysis, interpretation of data, revision for intellectual content, and final approval of the completed article. All authors contributed to the article and approved the submitted version.

## Funding

This work was supported by the National Research Foundation of Korea and funded by a grant from the Korean Government (No. 2021R1C1C1006794).

## Conflict of interest

Authors HC and SS are employed by INGRADIENT Inc. (Seoul, Republic of Korea). The remaining authors declare that the research was conducted in the absence of any commercial or financial relationships that could be construed as a potential conflict of interest.

## Publisher's note

All claims expressed in this article are solely those of the authors and do not necessarily represent those of their affiliated organizations, or those of the publisher, the editors and the reviewers. Any product that may be evaluated in this article, or claim that may be made by its manufacturer, is not guaranteed or endorsed by the publisher.

## Abbreviations

DSC: dice similarity coefficient
CT: computed tomography
CNN: convolutional neural networks
BW: body weight
BCS: body condition score
UNETR: u-net transformer.
